# The wrapped Rama distribution

**DOI:** 10.1038/s41598-024-83436-x

**Published:** 2024-12-30

**Authors:** William Bell, Saralees Nadarajah

**Affiliations:** Department of Mathematics, University of Manchester, Manchester, M13 9PL UK

**Keywords:** Circular data, Maximum likelihood, Trigonometric moments, Scientific data, Software, Statistics

## Abstract

A new one-parameter distribution is proposed for circular data based on wrapping. Most distributions constructed via wrapping do not yield elementary expressions for their mathematical properties. Yet the new distribution yields elementary expressions for all of its mathematical properties. Better fits of the new distribution over the three-parameter distribution due to Jones and Pewsey^10^ and six other wrapped distributions including four that have two parameters each are shown for at least two data sets. Better fits were assessed in terms of probability plots, density plots, values of Akaike information criterion and values of Bayesian information criterion.

## Introduction

Circular data are measurements that pertain to angular quantities, and are often expressed in units of degrees or radians. Examples of circular data can be found in many scientific disciplines such as astronomy (stellar motion tracking), biology (animal migration), engineering (wind direction analysis) and medicine (bone fracture orientation).

There have been many models proposed for circular data. Fisher^7^, Jones^9^, Ley and Verdebout^14^, Pewsey and Garcia-Portugues^18^and Ley et al. (2021) provide excellent reviews. With over 150 Google citations, perhaps the most popular model is that due to Jones and Pewsey^10^. Its probability density function is

1 $$\begin{aligned} \displaystyle f(x) = \frac{\left[ \textrm{cosh} (\kappa \psi ) + \textrm{sinh} (\kappa \psi ) \cos (x - \mu ) \right] ^{\frac{1}{\psi }}}{2 \pi P_{\frac{1}{\psi }} \left( \textrm{cosh} (\kappa \psi ) \right) } \end{aligned}$$

for $0 \le x < 2 \pi$, $0 \le \mu < 2 \pi$, $\kappa> 0$ and $-\infty< \psi < \infty$, where $P_a (x)$ denotes the Legendre function of the first kind defined by

2 $$\begin{aligned} \displaystyle P_a (x) = \ _2F_1 \left( -a, a + 1; 1; \frac{1 - x}{2} \right) , \end{aligned}$$

where $_2F_1 \left( a, b; c; z \right)$ denotes the Gauss hypergeometric function defined by

3 $$\begin{aligned} \displaystyle _2F_1 \left( a, b; c; z \right) = \sum _{k = 0}^\infty \frac{(a)_k (b)_k}{(c)_k} \frac{z^k}{k!}, \end{aligned}$$

where $(d)_k = d (d + 1) \cdots (d + k - 1)$ denotes the ascending factorial. We shall refer to the distribution given by (1) as the Jones-Pewsey distribution.

The aim of this paper is to propose a new one-parameter distribution for circular data constructed via the wrapping method that: i) gives elementary expressions for all its properties including the probability density function, cumulative distribution function, trigonometric moments and shape properties; most distributions constructed via the wrapping method are not elementary, examples include the wrapped normal and wrapped Cauchy distributions; ii) gives better fits than other distributions having more parameters, in particular the Jones-Pewsey distribution, for two data sets; iii) is easier to fit because of its elementary probability density function; the special function in (1) is not a standard special function, and so in-built routines for it are not so widely available.

The wrapping method is the most popular method for generating distributions for circular data. It can be described as follows. Suppose *g* is a valid probability density function. If *g* is defined on the positive real line then the probability density function of the wrapped distribution is

4 $$\begin{aligned} \displaystyle f (\theta ) = \sum _{k = 0}^\infty g \left( \theta + 2 k \pi \right) \end{aligned}$$

for $0 \le \theta < 2\pi$. If *g* is defined on the entire real line then the probability density function of the wrapped distribution is

5 $$\begin{aligned} \displaystyle f (\theta ) = \sum _{k = -\infty }^\infty g \left( \theta + 2 k \pi \right) \end{aligned}$$

for $$0 \le \theta < 2\pi$$.

There are over fifty papers proposing circular distributions using the method of wrapping. We mention^20^, Joshi and Jose^11^and Yilmaz and Bicer^24^as examples. Roy and Adnan^20^proposed a two-parameter wrapped weighted exponential distribution and compared its fit versus the wrapped normal distribution which has two parameters. Joshi and Jose^11^proposed a one-parameter wrapped Lindley distribution and compared its fit versus two other wrapped distributions each having one parameter. Yilmaz and Bicer^24^ proposed a two-parameter transmuted wrapped exponential distribution and compared its fit versus two other wrapped distributions each having one parameter. We are not aware of any wrapped distribution proposed in the literature whose fit has been compared to circular distributions having more parameters; especially, flexible distributions like the one in (1).

The contents of this paper are organized as follows. Section 2 proposes the new distribution. Section 3 derives several mathematical properties of the distribution. Estimation by method of maximum likelihood is considered in Section 4. Finite sample performance of the method of maximum likelihood is investigated by simulation in Section 5. Superior performance of the distribution over seven other distributions (five of which have more parameters) for two data sets is shown in Section 6. Some concluding remarks are given in Section 7. All computations in the paper were performed using the R software^19^. The R codes are given in a supplementary file.

## The new distribution

In this section, we propose a new distribution for circular data. A continuous random variable *Y*is said to follow the Rama distribution^22^ with shape parameter $\lambda> 0$ if its probability density and cumulative distribution functions are

6 $$\begin{aligned} \displaystyle f_Y \left( y\right) = \frac{\lambda ^4}{\lambda ^3+6}\left( 1+y^3\right) e^{-\lambda y} \end{aligned}$$

and

7 $$\begin{aligned} \displaystyle F_Y\left( y\right) = 1 - \left[ 1+\frac{\lambda y}{\lambda ^3+6}\left( {\lambda ^2 y^2 + 3\lambda y +6}\right) \right] e^{-\lambda y}, \end{aligned}$$

respectively, for $y> 0$ and $\lambda> 0$.

The Rama distribution has received various applications. Some recent applications include modeling diagnosis times of patients infected with hepatitis B^5^, modeling the strength of carbon fibers^6^, modeling the strength of aircraft windows^2^, modeling the strength of glass and carbon fibers^16^, modeling diagnosis times of patients infected with blood cancer^17^.

Let $\Theta = Y$ mod $2\pi$. Then $\Theta$ is said to have wrapped Rama distribution. By (4) and (5), the probability density function of $\Theta$ can be derived as

8 $$\begin{aligned} \displaystyle f_\Theta \left( \theta \right)&= \displaystyle \sum _{m=0}^{\infty }f(\theta +2m\pi ) = \frac{\lambda ^4}{\lambda ^3+6}\sum _{m=0}^{\infty }\left[ 1+\left( \theta +2m\pi \right) ^3\right] e^{-\lambda \left( \theta +2m\pi \right) }\nonumber \\&=\displaystyle \frac{\lambda ^4}{\lambda ^3+6}e^{-\lambda \theta } \Bigg [\sum _{m=0}^{\infty }e^{-2\lambda m \pi } + \sum _{m=0}^{\infty }\left( \theta +2m\pi \right) ^3e^{-2\lambda m \pi }\Bigg ] \nonumber \\&= \displaystyle \frac{\lambda ^4}{\lambda ^3+6}e^{-\lambda \theta } \Bigg [\left( \theta ^3+1\right) \sum _{m=0}^{\infty }e^{-2\lambda m \pi } + 6\pi \theta ^2\sum _{m=0}^{\infty }me^{-2\lambda m \pi } \nonumber \\&\qquad \displaystyle + 12\pi ^2\theta \sum _{m=0}^{\infty }m^2e^{-2\lambda m \pi } + 8\pi ^3\sum _{m=0}^{\infty }m^3e^{-2\lambda m \pi }\Bigg ] \nonumber \\&= \displaystyle \frac{\lambda ^4}{\lambda ^3+6}e^{-\lambda \theta } \Bigg [\frac{\theta ^3+1}{1-e^{-2\lambda \pi }} + \frac{6\pi \theta ^2e^{-2\lambda \pi }}{\left( 1-e^{-2\lambda \pi }\right) ^2} + \frac{12\pi ^2\theta e^{-2\lambda \pi }\left( e^{-2\lambda \pi }+1\right) }{\left( 1-e^{-2\lambda \pi }\right) ^3}\nonumber \\&\qquad \displaystyle +\frac{8\pi ^3 e^{-2\lambda \pi }\left( e^{-4\lambda \pi }+4e^{-2\lambda \pi }+1\right) }{\left( 1-e^{-2\lambda \pi }\right) ^4}\Bigg ]\nonumber \\&= \displaystyle A e^{-\lambda \theta } \left( \theta ^3 + B \theta ^2 + C \theta + D \right) \end{aligned}$$

for $$0 \le \theta < 2 \pi$$, where

9 $$\begin{aligned} \displaystyle A = \frac{\lambda ^4}{\left( \lambda ^3+6 \right) \left( 1-e^{-2\lambda \pi } \right) }, \end{aligned}$$

10 $$\begin{aligned} \displaystyle B = \frac{6\pi e^{-2\lambda \pi }}{1-e^{-2\lambda \pi }}, \end{aligned}$$

11 $$\begin{aligned} \displaystyle C = \frac{2\pi \left( e^{-2\lambda \pi }+1\right) B}{1-e^{-2\lambda \pi }} \end{aligned}$$

and

12 $$\begin{aligned} \displaystyle D = 1 + \frac{8\pi ^3 e^{-2 \lambda \pi } \left( e^{-4\lambda \pi }+4e^{-2\lambda \pi }+1\right) }{\left( 1-e^{-2\lambda \pi }\right) ^3}. \end{aligned}$$

## Mathematical properties

Let $\Theta$ denote a random variable having the probability density function (8). In this section, we derive several mathematical properties of $\Theta$, including shape properties of its probability density function, cumulative distribution function and trigonometric moments.

### Shape

The derivative of $\log f_\Theta \left( \theta \right)$ with respect to $\theta$ is

13 $$\begin{aligned} \displaystyle \frac{d \log f_\Theta \left( \theta \right) }{d \theta } = -\lambda + \frac{3 \theta ^2 + 2 B\theta + C}{\theta ^3 + B \theta ^2 + C \theta + D}. \end{aligned}$$

So, the critical points of $f_\Theta \left( \theta \right)$ are the roots of the cubic equation

14 $$\begin{aligned} \displaystyle \alpha \theta ^3 + \beta \theta ^2 + \gamma \theta + \delta = 0, \end{aligned}$$

where $\alpha = \lambda$, $\beta = \lambda B - 3$, $\gamma = \lambda C - 2 B$ and $\delta = \lambda D - C = 0$. The three possible roots of (14) are

15 $$\begin{aligned} \displaystyle \theta _k = -\frac{1}{3 \alpha } \left( \beta + \xi ^k C + \frac{\Delta _0}{\xi ^k C} \right) \end{aligned}$$

for $$k = 0, 1, 2$$, where

16 $$\begin{aligned}&\displaystyle \Delta _0 = \beta ^2 - 3 \alpha \gamma ,\end{aligned}$$

17 $$\begin{aligned}&\displaystyle \Delta _1 = 2 \beta ^3 - 9 \alpha \beta \gamma + 27 \alpha ^2 \delta ,\end{aligned}$$

18 $$\begin{aligned}&\displaystyle C = \left[ \frac{\Delta _1 \pm \sqrt{\Delta _1^2 - 4 \Delta _0^3}}{2} \right] ^{\frac{1}{3}} \end{aligned}$$

and $$\xi = \frac{-1 + \sqrt{-3}}{2}$$.

**Fig. 1:**
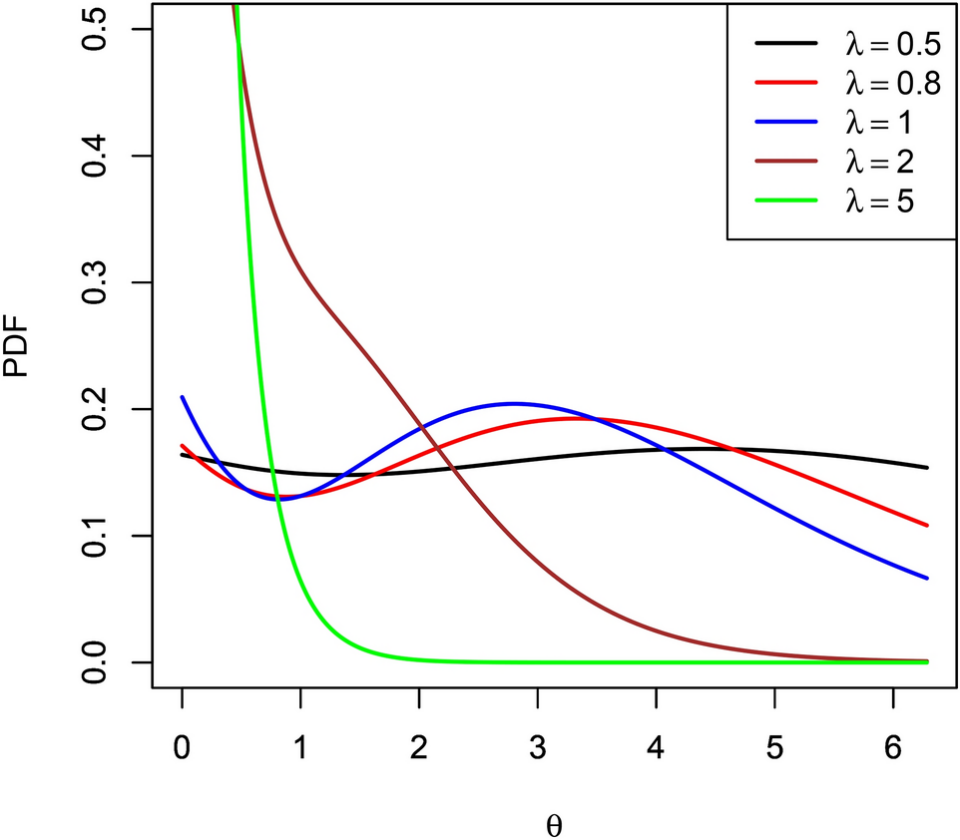
Possible shapes of the probability density function (8).

Possible shapes of $f_\Theta \left( \theta \right)$ are shown in Figure 1. If all of the roots in (15) are real and belong to $(0, 2 \pi ]$ then $f_\Theta \left( \theta \right)$ can have either a mode followed by an anti-mode which is followed by a mode or an anti-mode followed by a mode which is followed by an anti-mode. Assuming for the sake of argument that $\theta _0< \theta _1 < \theta _2$, the former will occur if $\Omega \left( \theta _0 \right) < 0$, $\Omega \left( \theta _1 \right)> 0$ and $\Omega \left( \theta _2 \right) < 0$, where

19 $$\begin{aligned} \displaystyle \Omega (\theta ) = \frac{6 \theta + 2 B}{\theta ^3 + B \theta ^2 + C \theta + D} - \left( \frac{3 \theta ^2 + 2 B\theta + C}{\theta ^3 + B \theta ^2 + C \theta + D} \right) ^2. \end{aligned}$$

The latter will occur if $\Omega \left( \theta _0 \right)> 0$, $\Omega \left( \theta _1 \right) < 0$ and $\Omega \left( \theta _2 \right)> 0$. If only two of the roots in (15) are real and belong to $(0, 2 \pi ]$ then $f_\Theta \left( \theta \right)$ can have either a mode followed by an anti-mode or an anti-mode followed by a mode. Assuming for the sake of argument that the two roots are $\theta _0 < \theta _2$, the former will occur if $\Omega \left( \theta _0 \right) < 0$ and $\Omega \left( \theta _2 \right)> 0$. The latter will occur if $\Omega \left( \theta _0 \right)> 0$ and $\Omega \left( \theta _2 \right) < 0$. If only one of the roots in (15) is real and belongs to $(0, 2 \pi ]$ then $f_\Theta \left( \theta \right)$ can have either a mode or an anti-mode. Assuming for the sake of argument that the root is $\theta _1$, the former will occur if $\Omega \left( \theta _1 \right) < 0$. The latter will occur if $\Omega \left( \theta _1 \right)> 0$. If $\alpha \theta ^3 + \beta \theta ^2 + \gamma \theta + \delta < 0$ for all $\theta$ then $f_\Theta \left( \theta \right)$ will be a monotonic decreasing function of $\theta$. If $\alpha \theta ^3 + \beta \theta ^2 + \gamma \theta + \delta> 0$ for all $\theta$ then $f_\Theta \left( \theta \right)$ will be a monotonic increasing function of $\theta$.

### Cumulative distribution function

The cumulative distribution function corresponding to (8) is

20 $$\begin{aligned} \displaystyle F_\Theta \left( \theta \right) = \frac{A}{\lambda ^4} \left[ \gamma (4, \lambda \theta ) + B \lambda \gamma (3, \lambda \theta ) + C \lambda ^2 \gamma (2, \lambda \theta ) + D \lambda ^3 \gamma (1, \lambda \theta ) \right] \end{aligned}$$

for $$0 \le \theta < 2 \pi$$, where

21 $$\begin{aligned} \gamma (n, x) = (n - 1)! \left( 1 - e^{-x} \sum _{k = 0}^{n - 1} \frac{x^k}{k!} \right) . \end{aligned}$$

Random numbers from the wrapped Rama distribution can be generated straightforwardly by inverting (20). We used the unirootfunction in the R software^19^ to invert (20). The inversion took just a fraction of a second.

### Trigonometric moments

The *n*th trigonometric moment of $\Theta$ is

22 $$\begin{aligned}&\displaystyle m_n = E \left[ e^{\textrm{i} n \Theta } \right] = \frac{A}{\left( \lambda - \textrm{i} n \right) ^4} \Bigg [ \gamma \left( 4, 2 \left( \lambda - \textrm{i} n \right) \pi \right) + B \left( \lambda - \textrm{i} n \right) \gamma \left( 3, 2 \left( \lambda - \textrm{i} n \right) \pi \right) \nonumber \\&\qquad \displaystyle +C \left( \lambda - \textrm{i} n \right) ^2 \gamma \left( 2, 2 \left( \lambda - \textrm{i} n \right) \pi \right) + D \left( \lambda - \textrm{i} n \right) ^3 \gamma \left( 1, 2 \left( \lambda - \textrm{i} n \right) \pi \right) \Bigg ] \end{aligned}$$

for $n = 1, 2, \ldots$, where $\textrm{i} = \sqrt{-1}$. Straightforward but lengthy calculations show that (22) can be rewritten as

23 $$\begin{aligned} \displaystyle m_n = \frac{A}{\left( \lambda ^2 + n^2 \right) ^4} \Bigg [ \left( c_0 + c_2 n^2 + c_4 n^4 + c_6 n^6 \right) + \textrm{i} \left( d_1 n + d_3 n^2 + d_5 n^5 + d_7 n^7 \right) \Bigg ], \end{aligned}$$

where

24 $$\begin{aligned}&\displaystyle c_0 = \lambda ^{7} - 4 \frac{{\lambda }^{6}B \pi }{\left( \textrm{e}^{\pi \lambda } \right) ^{2}} - 4 \frac{{\lambda }^{7}B {\pi }^{2}}{\textrm{e}^{2 \pi \lambda }} - 2 \frac{\lambda ^{7}C \pi }{\textrm{e}^{2 \pi \lambda }} - 2 \frac{\lambda ^5 B}{\textrm{e}^{2 \pi \lambda }} - \frac{\lambda ^{6} C}{\textrm{e}^{2 \pi \lambda }} - 6 \frac{\lambda ^4}{\textrm{e}^{2 \pi \lambda }} - 12 \frac{\pi ^{2} \lambda ^{6}}{\textrm{e}^{2 \pi \lambda }} \nonumber \\&\qquad \displaystyle -8 \frac{\pi ^{3} \lambda ^7}{\textrm{e}^{2 \pi \lambda }} - 12 \frac{\pi \lambda ^5}{\textrm{e}^{2 \pi \lambda }} + 6 \lambda ^{4} + \lambda ^{6}C - \frac{\lambda ^7}{\textrm{e}^{2 \pi \lambda }} + 2 \lambda ^{5} B, \end{aligned}$$

25 $$\begin{aligned}&\displaystyle c_2 = -\frac{\lambda ^{4}C}{\textrm{e}^{2 \pi \lambda }} + 4 \frac{\lambda ^{3}B}{\textrm{e}^{2 \pi \lambda }} + 24 \frac{\pi \lambda ^{3}}{\textrm{e}^{2 \pi \lambda }} - 12 \frac{\pi ^{2} \lambda ^{4}}{\textrm{e}^{2 \pi \lambda }} - 24 \frac{\pi ^{3} \lambda ^5}{\textrm{e}^{2 \pi \lambda }} + \lambda ^{4}C - 3 \frac{\lambda ^{5}}{\textrm{e}^{2 \pi \lambda }} - 4 \lambda ^{3} B - 36 \lambda ^{2} \nonumber \\&\qquad \displaystyle +36 \frac{\lambda ^{2}}{\textrm{e}^{2 \pi \lambda }} + 3 \lambda ^{5} - 4 \frac{\lambda ^{4}B \pi }{\textrm{e}^{2 \pi \lambda }} - 6 \frac{\lambda ^{5} C \pi }{\textrm{e}^{2 \pi \lambda }} - 12 \frac{\lambda ^{5} B \pi ^{2}}{\textrm{e}^{2 \pi \lambda }}, \end{aligned}$$

26 $$\begin{aligned}&\displaystyle c_4 = \frac{\lambda ^{2}C}{\textrm{e}^{2 \pi \lambda }} + 6 \frac{\lambda B}{\textrm{e}^{2 \pi \lambda }} + 36 \frac{\pi \lambda }{\textrm{e}^{2 \pi \lambda }} + 12 \frac{\pi ^{2} \lambda ^{2}}{\textrm{e}^{2 \pi \lambda }} - 24 \frac{\pi ^{3} \lambda ^{3}}{\textrm{e}^{2 \pi \lambda }} - 3 \frac{\lambda ^3}{\textrm{e}^{2 \pi \lambda }} - \lambda ^{2}C - 6 \lambda B - \frac{6}{\textrm{e}^{2 \pi \lambda }} \nonumber \\&\qquad \displaystyle + 6 + 3 \lambda ^{3} - 6 \frac{C \pi \lambda ^3}{\textrm{e}^{2 \pi \lambda }} - 12 \frac{B \pi ^{2} \lambda ^{3}}{\textrm{e}^{2 \pi \lambda }} + 4 \frac{B \pi \lambda ^2}{\textrm{e}^{2 \pi \lambda }}, \end{aligned}$$

27 $$\begin{aligned} \displaystyle c_6 = 4 \frac{B \pi }{\textrm{e}^{2 \pi \lambda }} - 8 \frac{\pi ^{3} \lambda }{\textrm{e}^{2 \pi \lambda }} - \frac{\lambda }{\textrm{e}^{2 \pi \lambda }} + \frac{C}{\textrm{e}^{2 \pi \lambda }} + 12 \frac{\pi ^{2}}{\textrm{e}^{2 \pi \lambda }} + \lambda - C - 2 \frac{\lambda C \pi }{\textrm{e}^{2 \pi \lambda }} - 4 \frac{\lambda B \pi ^2}{\textrm{e}^{2 \pi \lambda }}, \end{aligned}$$

28 $$\begin{aligned}&\displaystyle d_1 = 2 \lambda ^{5} C - \frac{\lambda ^{6}}{\textrm{e}^{2 \pi \lambda }} + 6 \lambda ^{4} B - 6 \frac{\lambda ^{4}B}{\textrm{e}^{2 \pi \lambda }} - 2 \frac{\lambda ^{5}C}{\textrm{e}^{2 \pi \lambda }} - 24 \frac{\pi ^{2} \lambda ^{5}}{\textrm{e}^{2 \pi \lambda }} - 8 \frac{\pi ^3 \lambda ^6}{\textrm{e}^{2 \pi \lambda }} - 3 \frac{\pi \lambda ^4}{\textrm{e}^{2 \pi \lambda }} + 24 \lambda ^{3} \nonumber \\&\qquad \displaystyle -24 \frac{\lambda ^3}{\textrm{e}^{2 \pi \lambda }} + \lambda ^{6} - 8 \frac{\lambda ^{5} B \pi }{\textrm{e}^{2 \pi \lambda }} - 4 \frac{\lambda ^{6}B \pi ^{2}}{\textrm{e}^{2 \pi \lambda }} - 2 \frac{\lambda ^{6}C \pi }{\textrm{e}^{2 \pi \lambda }}, \end{aligned}$$

29 $$\begin{aligned}&\displaystyle d_3 = -3 \frac{\lambda ^4}{\textrm{e}^{2 \pi \lambda }} + 4 \lambda ^{3}C + 4 \lambda ^{2}B - 4 \frac{\lambda ^3 C}{\textrm{e}^{2 \pi \lambda }} - 4 \frac{\lambda ^{2}B}{\textrm{e}^{2 \pi \lambda }} - 24 \frac{\pi \lambda ^2}{\textrm{e}^{2 \pi \lambda }} - 48 \frac{\pi ^{2} \lambda ^3}{\textrm{e}^{2 \pi \lambda }} - 24 \frac{\pi ^{3} \lambda ^4}{\textrm{e}^{2 \pi \lambda }} - 24 \lambda \nonumber \\&\qquad \displaystyle +24 \frac{\lambda }{\textrm{e}^{2 \pi \lambda }} + 3 \lambda ^{4} - 6 \frac{\lambda ^{4} C \pi }{\textrm{e}^{2 \pi \lambda }} - 12 \frac{\lambda ^{4}B \pi ^2}{\textrm{e}^{2 \pi \lambda }} - 16 \frac{\lambda ^{3}B \pi }{\textrm{e}^{2 \pi \lambda }}, \end{aligned}$$

30 $$\begin{aligned}&\displaystyle d_5 = 2 \lambda C + 2 \frac{B}{\textrm{e}^{2 \pi \lambda }} - 3 \frac{\lambda ^2}{\textrm{e}^{2 \pi \lambda }} - 2 \frac{\lambda C}{\textrm{e}^{2 \pi \lambda }} - 24 \frac{\pi ^{2} \lambda }{\textrm{e}^{2 \pi \lambda }} - 24 \frac{\pi ^{3} \lambda ^2}{\textrm{e}^{2 \pi \lambda }} + 12 \frac{\pi }{\textrm{e}^{2 \pi \lambda }} + 3 \lambda ^{2} - 2 B \nonumber \\&\qquad \displaystyle -6 \frac{\lambda ^{2}C \pi }{\textrm{e}^{2 \pi \lambda }} - 12 \frac{\lambda ^{2} B \pi ^2}{\textrm{e}^{2 \pi \lambda }} - 8 \frac{\lambda B \pi }{\textrm{e}^{2 \pi \lambda }}, \end{aligned}$$

and

31 $$\begin{aligned} \displaystyle d_7 = 1 - 2 \frac{C \pi }{\textrm{e}^{2 \pi \lambda }} - 4 \frac{B \pi ^2}{\textrm{e}^{2 \pi \lambda }} - 8 \frac{\pi ^3}{\textrm{e}^{2 \pi \lambda }} - \frac{1}{\textrm{e}^{2 \pi \lambda }}. \end{aligned}$$

Hence, the mean angle, mean resultant, circular variance, circular skewness and circular kurtosis of $\Theta$ are

32 $$\begin{aligned} \displaystyle \mu = \arcsin \left[ \frac{d_1 + d_3 + d_5 + d_7}{\sqrt{\left( c_0 + c_2 + c_4 + c_6 \right) ^2 + \left( d_1 + d_3 + d_5 + d_7 \right) ^2}} \right] , \end{aligned}$$

33 $$\begin{aligned} \displaystyle \rho = \frac{A}{\left( \lambda ^2 + 1 \right) ^4} \sqrt{\left( c_0 + c_2 + c_4 + c_6 \right) ^2 + \left( d_1 + d_3 + d_5 + d_7 \right) ^2}, \end{aligned}$$

34 $$\begin{aligned} \displaystyle v = 1 - \frac{A}{\left( \lambda ^2 + 1 \right) ^4} \sqrt{\left( c_0 + c_2 + c_4 + c_6 \right) ^2 + \left( d_1 + d_3 + d_5 + d_7 \right) ^2}, \end{aligned}$$

35 $$\begin{aligned} \displaystyle \gamma _1 = \frac{A e^{-2 \mu }}{v^{\frac{3}{2}} \left( \lambda ^2 + 4 \right) ^4} \left( 2 d_1 + 4 d_3 + 32 d_5 + 128 d_7 \right) \end{aligned}$$

and

36 $$\begin{aligned} \displaystyle \gamma _2 = \frac{A e^{-2 \mu }}{\left( 1 - \rho ^2 \right) \left( \lambda ^2 + 4 \right) ^4} \left( c_0 + 4 c_2 + 16 c_4 + 64 c_6 \right) - \frac{\rho ^4}{1 - \rho ^2}, \end{aligned}$$

respectively.

## Maximum likelihood estimation

Suppose $\theta _1, \theta _2, \ldots , \theta _n$ is a random sample from (8) with $\lambda$ unknown. The log-likelihood function of $\lambda$ is

37 $$\begin{aligned} \displaystyle \log L(\lambda ) = n \log A - \lambda \sum _{i = 1}^n \theta _i + \sum _{i = 1}^n \log \left( \theta _i^3 + B \theta _i^2 + C \theta _i + D \right) . \end{aligned}$$

Its derivative with respect to $\lambda$ is

38 $$\begin{aligned} \displaystyle \frac{d \log L(\lambda )}{d \lambda } = \frac{n}{A} \frac{d A}{d \lambda } - \sum _{i = 1}^n \theta _i + \sum _{i = 1}^n \frac{\theta _i^3 + \frac{d B}{d \lambda } \theta _i^2 + \frac{d C}{d \lambda } \theta _i + \frac{d D}{d \lambda }}{\theta _i^3 + B \theta _i^2 + C \theta _i + D}, \end{aligned}$$

where

39 $$\begin{aligned} \displaystyle \frac{d A}{d \lambda } = 4 \frac{\lambda ^3}{\left( {\lambda }^{3}+6 \right) \left( 1-{e}^{-2 \lambda \pi } \right) } - 3 \frac{\lambda ^6}{\left( {\lambda }^{3}+6 \right) ^{2} \left( 1-{e}^{-2 \lambda \pi } \right) } - 2\frac{\lambda ^{4}{e}^{-2 \lambda \pi }\pi }{ \left( \lambda ^{3}+6 \right) \left( 1-{e}^{-2 \lambda \pi } \right) ^{2}}, \end{aligned}$$

40 $$\begin{aligned} \displaystyle \frac{d B}{d \lambda } = -\frac{12 \pi ^2 {e}^{-2 \lambda \pi }}{1-{e}^{-2 \lambda \pi }} - \frac{12 \pi ^2 {e}^{-4 \lambda \pi }}{\left( 1-{e}^{-2 \lambda \pi } \right) ^2}, \end{aligned}$$

41 $$\begin{aligned} \displaystyle \frac{d C}{d \lambda } = -\frac{24 \pi ^3 {e}^{-4 \lambda \pi }}{\left( 1-{e}^{-2 \lambda \pi } \right) ^2} - \frac{24 \pi ^3 \left( 1+{e}^{-2 \lambda \pi } \right) {e}^{-2 \lambda \pi }}{\left( 1-{e}^{-2 \lambda \pi } \right) ^2} - \frac{48 \pi ^3 \left( 1+{e}^{-2 \lambda \pi } \right) {e}^{-4 \lambda \pi }}{\left( 1-{e}^{-2 \lambda \pi } \right) ^3} \end{aligned}$$

and

42 $$\begin{aligned}&\displaystyle \frac{d D}{d \lambda } = -\frac{16 \pi ^4 \left( 1 + 4 {e}^{-2 \lambda \pi } + {e}^{-4 \lambda \pi } \right) {e}^{-2 \lambda \pi }}{\left( 1-{e}^{-2 \lambda \pi } \right) ^3} - \frac{32 \pi ^4 \left( 2 {e}^{-2 \lambda \pi } + {e}^{-4 \lambda \pi } \right) {e}^{-2 \lambda \pi }}{\left( 1-{e}^{-2 \lambda \pi } \right) ^3}\nonumber \\&\qquad \displaystyle -\frac{48 \pi ^4 \left( 1 + 4 {e}^{-2 \lambda \pi } + {e}^{-4 \lambda \pi } \right) {e}^{-4 \lambda \pi }}{\left( 1-{e}^{-2 \lambda \pi } \right) ^4}. \end{aligned}$$

The maximum likelihood estimate of $\lambda$, say $\widehat{\lambda }$, is the root of

43 $$\begin{aligned} \displaystyle \frac{n}{A} \frac{d A}{d \lambda } + \sum _{i = 1}^n \frac{\theta _i^3 + \frac{d B}{d \lambda } \theta _i^2 + \frac{d C}{d \lambda } \theta _i + \frac{d D}{d \lambda }}{\theta _i^3 + B \theta _i^2 + C \theta _i + D} = \sum _{i = 1}^n \theta _i \end{aligned}$$

for $\lambda$. (43) was solved numerically using the uniroot function in the R software to obtain the maximum likelihood estimate.

The second order derivative of (37) with respect to $\lambda$ is

44 $$\begin{aligned}&\displaystyle \frac{d^2 \log L(\lambda )}{d \lambda ^2} = -\frac{n}{A^2} \left( \frac{d A}{d \lambda } \right) ^2 + \frac{n}{A} \frac{d^2 A}{d \lambda ^2} + \sum _{i = 1}^n \theta _i + \sum _{i = 1}^n \frac{\theta _i^3 + \frac{d^2 B}{d \lambda ^2} \theta _i^2 + \frac{d^2 C}{d \lambda ^2} \theta _i + \frac{d^2 D}{d \lambda ^2}}{\theta _i^3 + B \theta _i^2 + C \theta _i + D}\nonumber \\&\qquad \displaystyle -\sum _{i = 1}^n \left( \frac{\theta _i^3 + \frac{d B}{d \lambda } \theta _i^2 + \frac{d C}{d \lambda } \theta _i + \frac{d D}{d \lambda }}{\theta _i^3 + B \theta _i^2 + C \theta _i + D} \right) ^2, \end{aligned}$$

where

45 $$\begin{aligned}&\displaystyle \frac{d^2 A}{d \lambda ^2} = 12 \frac{{\lambda }^{2}}{\left( {\lambda }^{3}+6 \right) \left( 1-{e}^{-2 \lambda \pi } \right) } - 30 \frac{{\lambda }^{5}}{\left( {\lambda }^{3}+6 \right) ^{2} \left( 1-{e}^{-2 \lambda \pi } \right) } - 16 \frac{{e}^{-2 \lambda \pi }{\lambda }^{3}\pi }{\left( {\lambda }^{3}+6 \right) \left( 1-{e}^{-2 \lambda \pi } \right) ^{2}}\nonumber \\&\qquad \qquad \displaystyle +18 \frac{{\lambda }^{8}}{ \left( {\lambda }^{3}+6 \right) ^{3} \left( 1-{e}^{-2 \lambda \pi } \right) } + 12 \frac{{\lambda }^{6}{e}^{-2 \lambda \pi }\pi }{\left( {\lambda }^{3}+6 \right) ^{2} \left( 1-{e}^{-2 \lambda \pi } \right) ^{2}} \nonumber \\&\qquad \qquad \displaystyle +8 \frac{{\lambda }^{4} {e}^{-4 \lambda \pi } {\pi }^{2}}{\left( {\lambda }^{3}+6 \right) \left( 1-{e}^{-2 \lambda \pi } \right) ^{3}} + 4 \frac{{\lambda }^{4}{e}^{-2 \lambda \pi }{\pi }^{2}}{ \left( {\lambda }^{3}+6 \right) \left( 1-{e}^{-2 \lambda \pi } \right) ^{2}}, \end{aligned}$$

46 $$\begin{aligned} \displaystyle \frac{d^2 B}{d \lambda ^2} = 24 \frac{\pi ^{3} {e}^{-2 \lambda \pi }}{1-{e}^{-2 \lambda \pi }} + 72 \frac{\pi ^{3} {e}^{-4 \lambda \pi }}{\left( 1-{e}^{-2 \lambda \pi } \right) ^2} + 48 \frac{\pi ^{3} {e}^{-6 \lambda \pi }}{\left( 1-{e}^{-2 \lambda \pi } \right) ^3}, \end{aligned}$$

47 $$\begin{aligned}&\displaystyle \frac{d^2 C}{d \lambda ^2} = 144 \frac{\pi ^4 {e}^{-4 \lambda \pi }}{\left( 1-{e}^{-2 \lambda \pi } \right) ^2} + 192 \frac{\pi ^4 {e}^{-6 \lambda \pi }}{\left( 1-{e}^{-2 \lambda \pi } \right) ^3} + 48 \frac{\pi ^4 {e}^{-2 \lambda \pi }}{\left( 1-{e}^{-2 \lambda \pi } \right) ^2} \nonumber \\&\qquad \qquad \displaystyle +288 \frac{\pi ^4 \left( 1 + {e}^{-2 \lambda \pi } \right) {e}^{-4 \lambda \pi }}{\left( 1-{e}^{-2 \lambda \pi } \right) ^3} + 288 \frac{\pi ^4 \left( 1 + {e}^{-2 \lambda \pi } \right) {e}^{-6 \lambda \pi }}{\left( 1-{e}^{-2 \lambda \pi } \right) ^4} \end{aligned}$$

and

48 $$\begin{aligned}&\displaystyle \frac{d^2 D}{d \lambda ^2} = 32 \frac{\pi ^5 \left( 1 + 4 {e}^{-2 \lambda \pi } + {e}^{-4 \lambda \pi } \right) {e}^{-2 \lambda \pi }}{\left( 1-{e}^{-2 \lambda \pi } \right) ^3} + 128 \frac{\pi ^5 \left( 2 + {e}^{-2 \lambda \pi } \right) {e}^{-2 \lambda \pi }}{\left( 1-{e}^{-2 \lambda \pi } \right) ^3} \nonumber \\&\qquad \qquad \displaystyle +288 \frac{\pi ^5 \left( 1 + 4 {e}^{-2 \lambda \pi } + {e}^{-4 \lambda \pi } \right) {e}^{-4 \lambda \pi }}{\left( 1-{e}^{-2 \lambda \pi } \right) ^4} + 128 \frac{\pi ^5 \left( 1 + {e}^{-2 \lambda \pi } \right) {e}^{-4 \lambda \pi }}{\left( 1-{e}^{-2 \lambda \pi } \right) ^3} \nonumber \\&\qquad \qquad \displaystyle +384 \frac{\pi ^5 \left( 2 + {e}^{-2 \lambda \pi } \right) {e}^{-6 \lambda \pi }}{\left( 1-{e}^{-2 \lambda \pi } \right) ^4} + 384 \frac{\pi ^5 \left( 1 + 4 {e}^{-2 \lambda \pi } + {e}^{-4 \lambda \pi } \right) {e}^{-6 \lambda \pi }}{\left( 1-{e}^{-2 \lambda \pi } \right) ^5}. \end{aligned}$$

Hence, assuming asymptotic normality of $\widehat{\lambda }$ holds, an approximate $100(1 - \alpha )$ percent confidence interval for $\lambda$ is

49 $$\begin{aligned} \displaystyle \widehat{\lambda } \pm z_{\frac{\alpha }{2}} \left. \left\{ \frac{d^2 \log L(\lambda )}{d \lambda ^2} \right| _{\lambda = \widehat{\lambda }} \right\} ^{-\frac{1}{2}}, \end{aligned}$$

where $z_{\frac{\alpha }{2}}$ denotes the $100 \left( 1 - \frac{\alpha }{2} \right)$ percentile of a standard normal random variable.

## Simulation study

In this section, we conduct a simulation study to check the finite sample performance of $\widehat{\lambda }$ in Section 4. The finite sample performance is checked with respect to bias, mean squared error, coverage length and coverage probability^4^. We used the following scheme:

- set $$\lambda = 1$$ and $$n = 10$$;
- simulate a random sample $\left\{ u_1, \ldots , u_n \right\}$ from the uniform [0, 1] distribution;
- solve
  50 $$\begin{aligned} \displaystyle \frac{A}{\lambda ^4} \left[ \gamma \left( 4, \lambda \theta _i\right) + B \lambda \gamma \left( 3, \lambda \theta _i\right) + C \lambda ^2 \gamma \left( 2, \lambda \theta _i\right) + D \lambda ^3 \gamma \left( 1, \lambda \theta _i\right) \right] = u_i \end{aligned}$$
  for $\theta _i$ for $i = 1, \ldots , n$, using the uniroot function in the R software, where *A*, *B*, *C* and *D* are as given in Section 2;
- compute the maximum likelihood estimate, say $\widehat{\lambda }$, by solving
  51 $$\begin{aligned} \displaystyle \frac{n}{A} \frac{d A}{d \lambda } + \sum _{i = 1}^n \frac{\theta _i^3 + \frac{d B}{d \lambda } \theta _i^2 + \frac{d C}{d \lambda } \theta _i + \frac{d D}{d \lambda }}{\theta _i^3 + B \theta _i^2 + C \theta _i + D} = \sum _{i = 1}^n \theta _i \end{aligned}$$
  for $\lambda$, once again the uniroot function in the R software was used for solving;
- repeat steps b) to d) one thousand times, obtaining one thousand estimates for $\widehat{\lambda }$, say $\widehat{\lambda }_1, \ldots , \widehat{\lambda }_{1000}$;
- compute the bias as
  52 $$\begin{aligned} \displaystyle \textrm{bias}_n \left( \widehat{\lambda } \right) = \frac{1}{1000} \sum _{j = 1}^{1000} \left( \widehat{\lambda }_j - 1 \right) ; \end{aligned}$$
- compute the mean squared error as
  53 $$\begin{aligned} \displaystyle \textrm{mse}_n \left( \widehat{\lambda } \right) = \frac{1}{1000} \sum _{j = 1}^{1000} \left( \widehat{\lambda }_j - 1 \right) ^2; \end{aligned}$$
- compute the coverage length as
  54 $$\begin{aligned} \displaystyle \textrm{CL}_n \left( \widehat{\lambda } \right) = \frac{z_{0.975}}{500} \sum _{j = 1}^{1000} \left. \left\{ \frac{d^2 \log L(\lambda )}{d \lambda ^2} \right| _{\lambda = \widehat{\lambda }_j} \right\} ^{-\frac{1}{2}}, \end{aligned}$$
  where $\frac{d^2 \log L(\lambda )}{d \lambda ^2}$ is given by (44);
- compute the coverage probability as
  55 $$\begin{aligned}&\displaystyle \textrm{CP}_n \left( \widehat{\lambda } \right) = \frac{1}{1000} \sum _{j = 1}^{1000} I \Bigg \{ \widehat{\lambda }_j - z_{0.975} \left. \left\{ \frac{d^2 \log L(\lambda )}{d \lambda ^2} \right| _{\lambda = \widehat{\lambda }_j} \right\} ^{-\frac{1}{2}}< 1 \nonumber \\&\qquad \qquad \qquad \qquad \qquad \displaystyle < \widehat{\lambda }_j + z_{0.975} \left. \left\{ \frac{d^2 \log L(\lambda )}{d \lambda ^2} \right| _{\lambda = \widehat{\lambda }_j} \right\} ^{-\frac{1}{2}} \Bigg \}, \end{aligned}$$
  where $I \left\{ \cdot \right\}$ denotes the indicator function;
- repeat steps b) to i) for $n = 11, 12, \ldots , 100$.

**Fig. 2:**
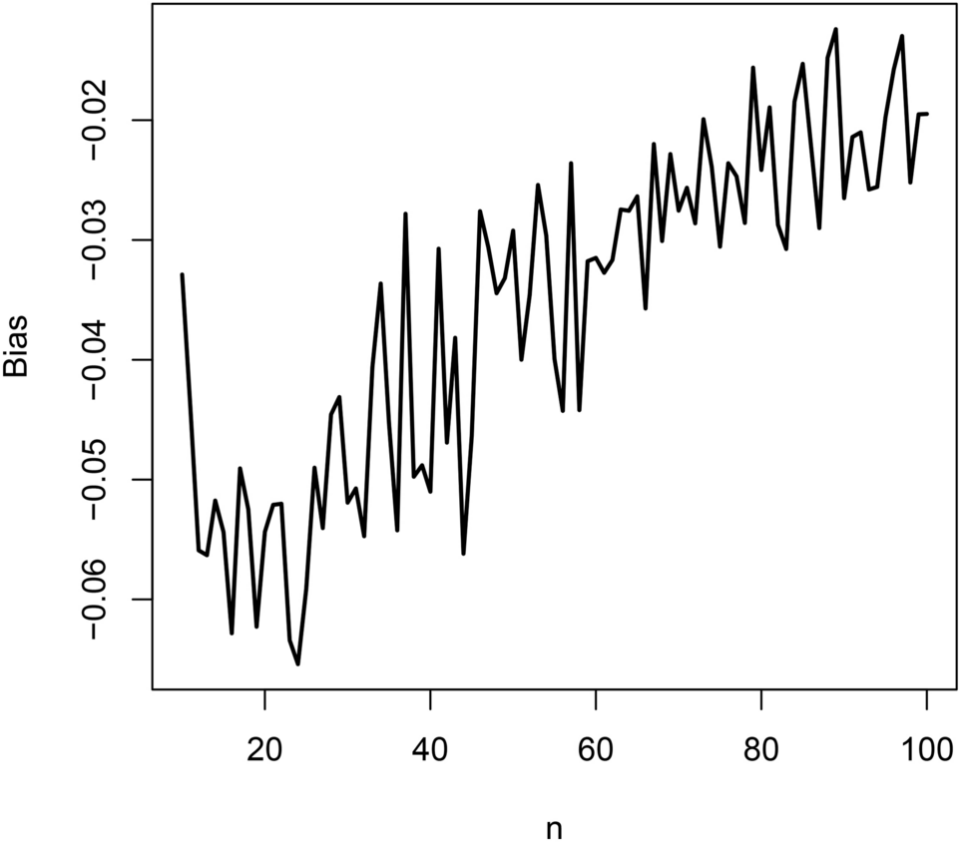
Biases versus $$n = 10, 11, \ldots , 100$$.

**Fig. 3:**
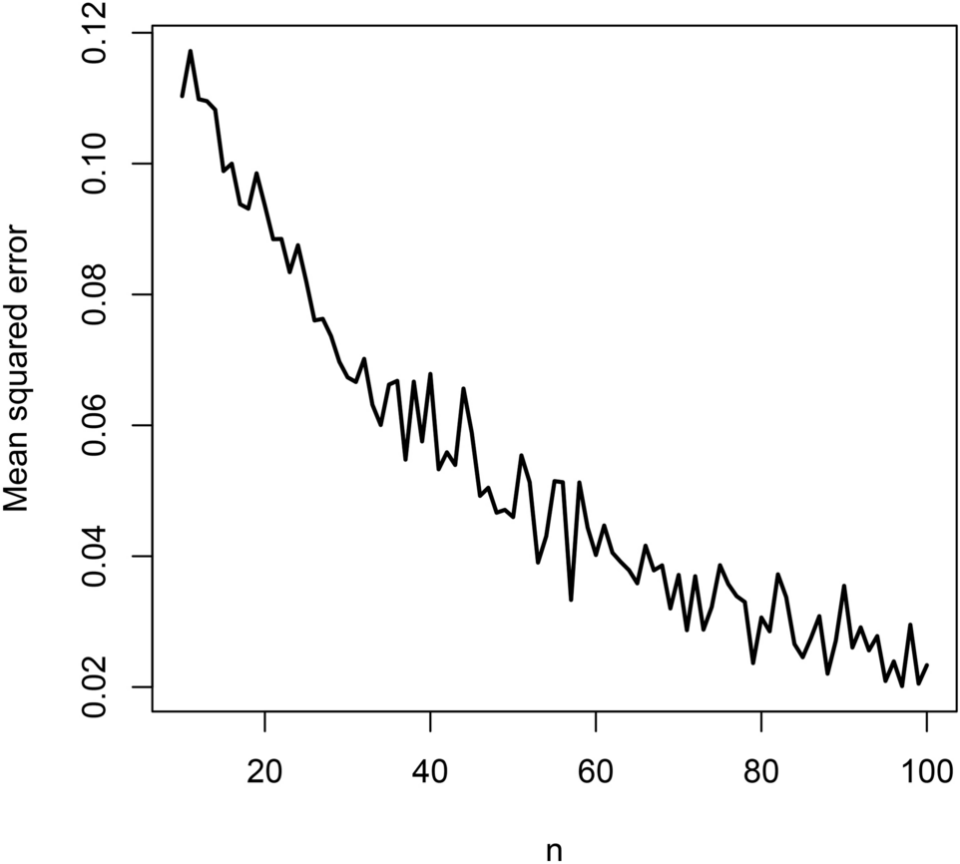
Mean squared errors versus $n = 10, 11, \ldots , 100$.

**Fig. 4:**
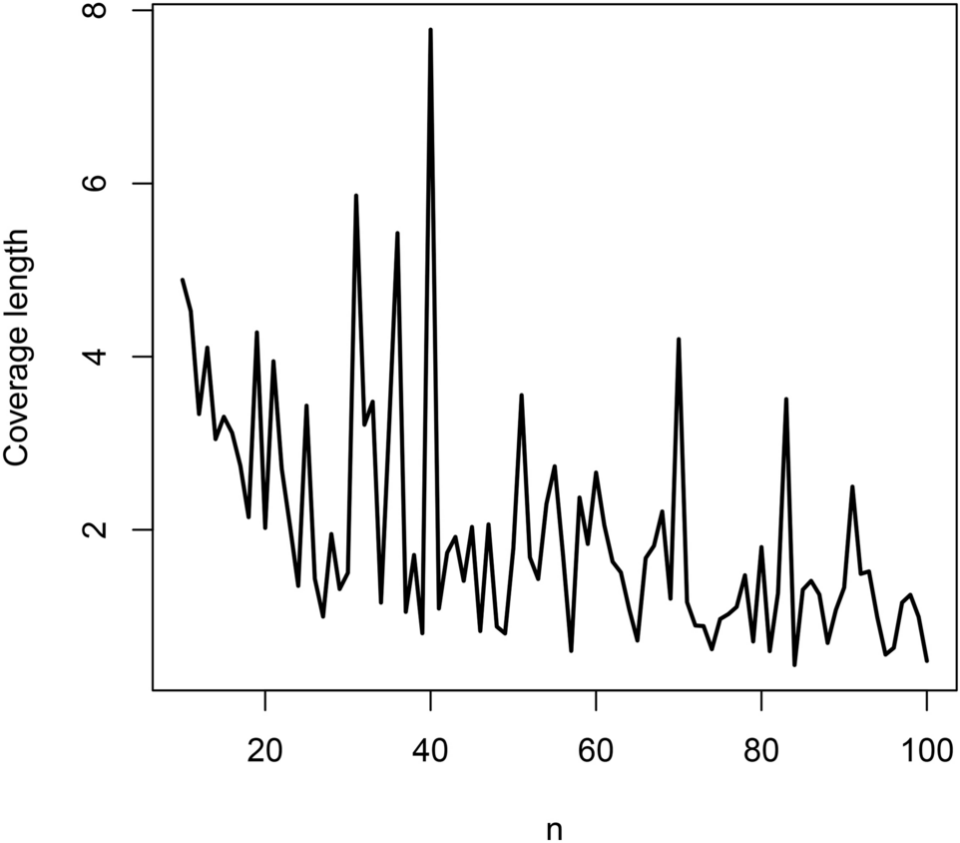
Coverage lengths versus $n = 10, 11, \ldots , 100$.

**Fig. 5:**
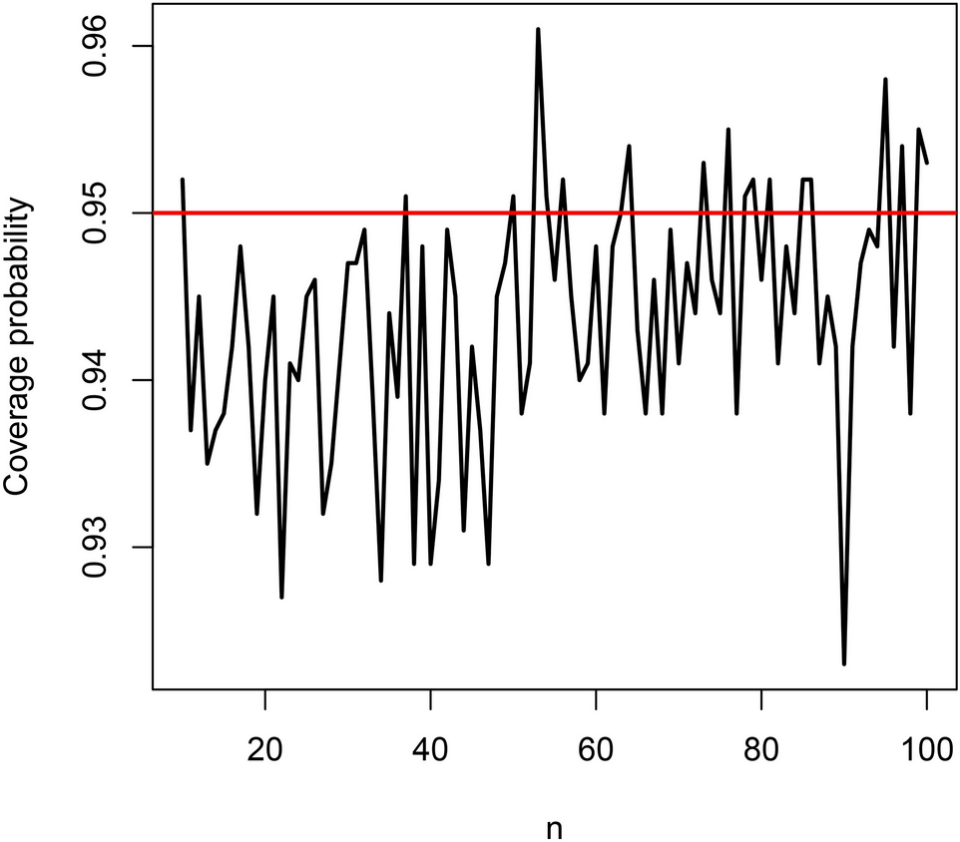
Coverage probabilities versus $n = 10, 11, \ldots , 100$.

The biases $\textrm{bias}_{10} \left( \widehat{\lambda } \right) , \ldots , \textrm{bias}_{100} \left( \widehat{\lambda } \right)$ are plotted in Figure 2. The mean squared errors $\textrm{mse}_{10} \left( \widehat{\lambda } \right) , \ldots , \textrm{mse}_{100} \left( \widehat{\lambda } \right)$ are plotted in Figure 3. The coverage lengths $\textrm{CL}_{10} \left( \widehat{\lambda } \right) , \ldots , \textrm{CL}_{100} \left( \widehat{\lambda } \right)$ are plotted in Figure 4. The coverage probabilities $\textrm{CP}_{10} \left( \widehat{\lambda } \right) , \ldots , \textrm{CP}_{100} \left( \widehat{\lambda } \right)$ are plotted in Figure 5. The horizontal line in Figure 5 corresponds to the nominal level of 0.95.

The biases are negative and increase from $-0.06$ to $-0.02$ as *n* approaches 100. They appear reasonably close to zero for all $n \ge 80$. The mean squared errors decrease from 0.12 to 0.02 as *n* approaches 100. They appear reasonably close to zero also for all $n \ge 80$. The coverage lengths decrease from about 5 to 1 as *n* approaches 100. They appear reasonably accurate for all $n \ge 80$. The coverage probabilities approach the nominal level as *n* approaches 100. They appear reasonably close to the nominal level for all $n \ge 80$.

The observations noted are for the particular initial value $\lambda = 1$. But the same observations held for a wide range of other values of $\lambda$. In particular, the magnitude of biases always decreased to zero with increasing *n*, the mean squared errors always decreased to zero with increasing *n*, the coverage lengths always decreased with increasing *n* and the coverage probabilities always approached the nominal level with increasing *n*.

Hence, the maximum likelihood estimates of the wrapped Rama distribution can be considered to behave according to the large sample theory of maximum likelihood estimation for all $n \ge 80$. In particular, both point and interval estimates of $\lambda$ satisfy the large sample theory, enabling construction of confidence intervals, likelihood ratio tests and other tests of hypothesis among others.

## Data applications

In this section, we illustrate flexibility of the wrapped Rama (WR) distribution using two real data sets. We compare its fit versus the two-parameter Wrapped Normal (WN) distribution, the two-parameter Wrapped Cauchy (WC) distribution, the one-parameter Wrapped Exponential (TWE) distribution due to Jammalamadaka and Kozubowski^8^, the two-parameter Transmuted Wrapped Exponential (TWE) distribution due to Yilmaz and Bicer^24^, the one-parameter Wrapped Lindley (WL) distribution due to Joshi and Jose^11^, the two-parameter Wrapped Quasi Lindley (WQL) distribution due to Al-Khazaleh and Al-Khazaleh^3^ and the three-parameter Jones-Pewsey (JP) distribution.

**Fig. 6:**
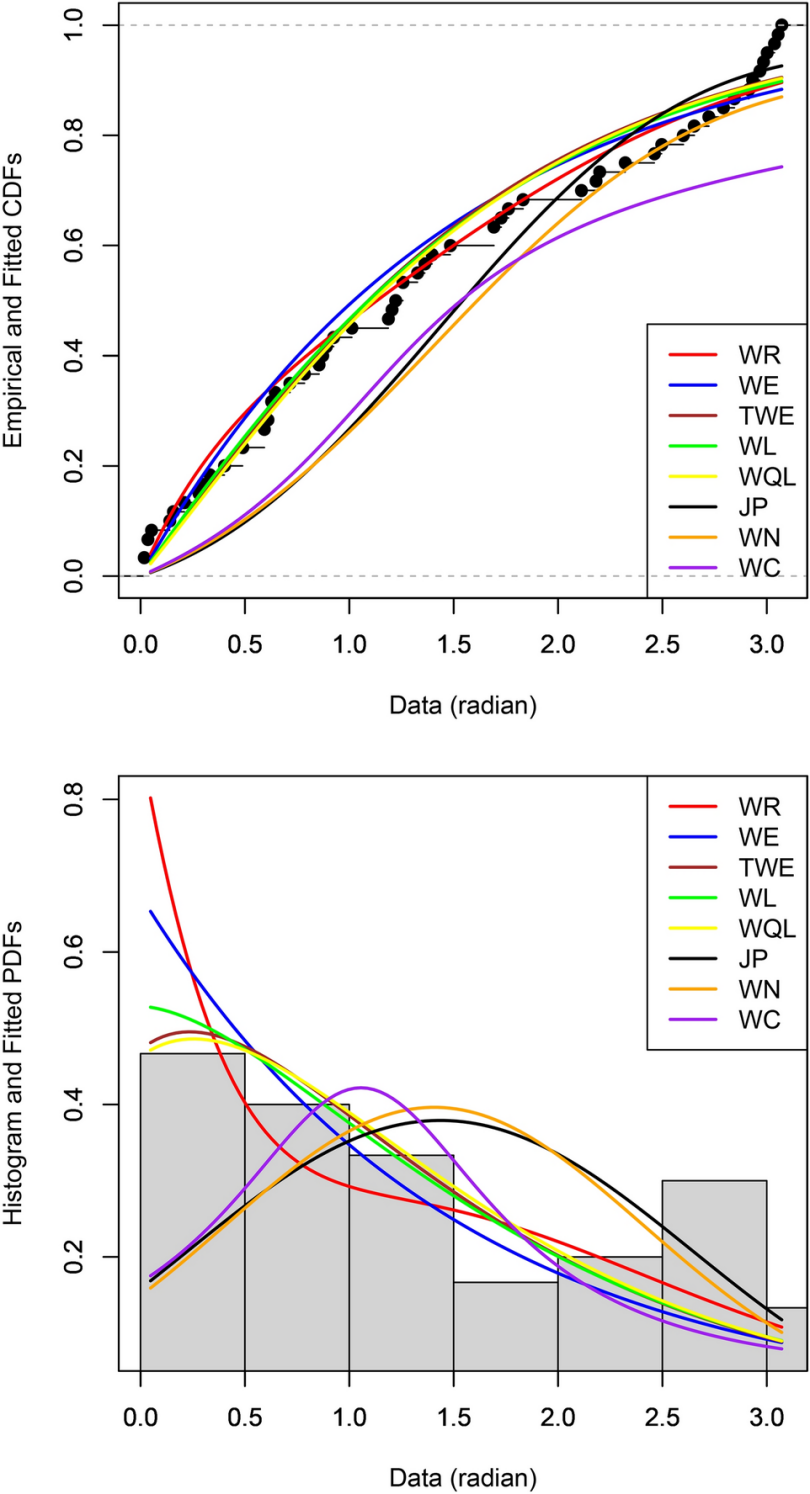
Probability plot (top) and density plot (bottom) of the fitted distributions for the feldspar laths data set.

**Fig. 7:**
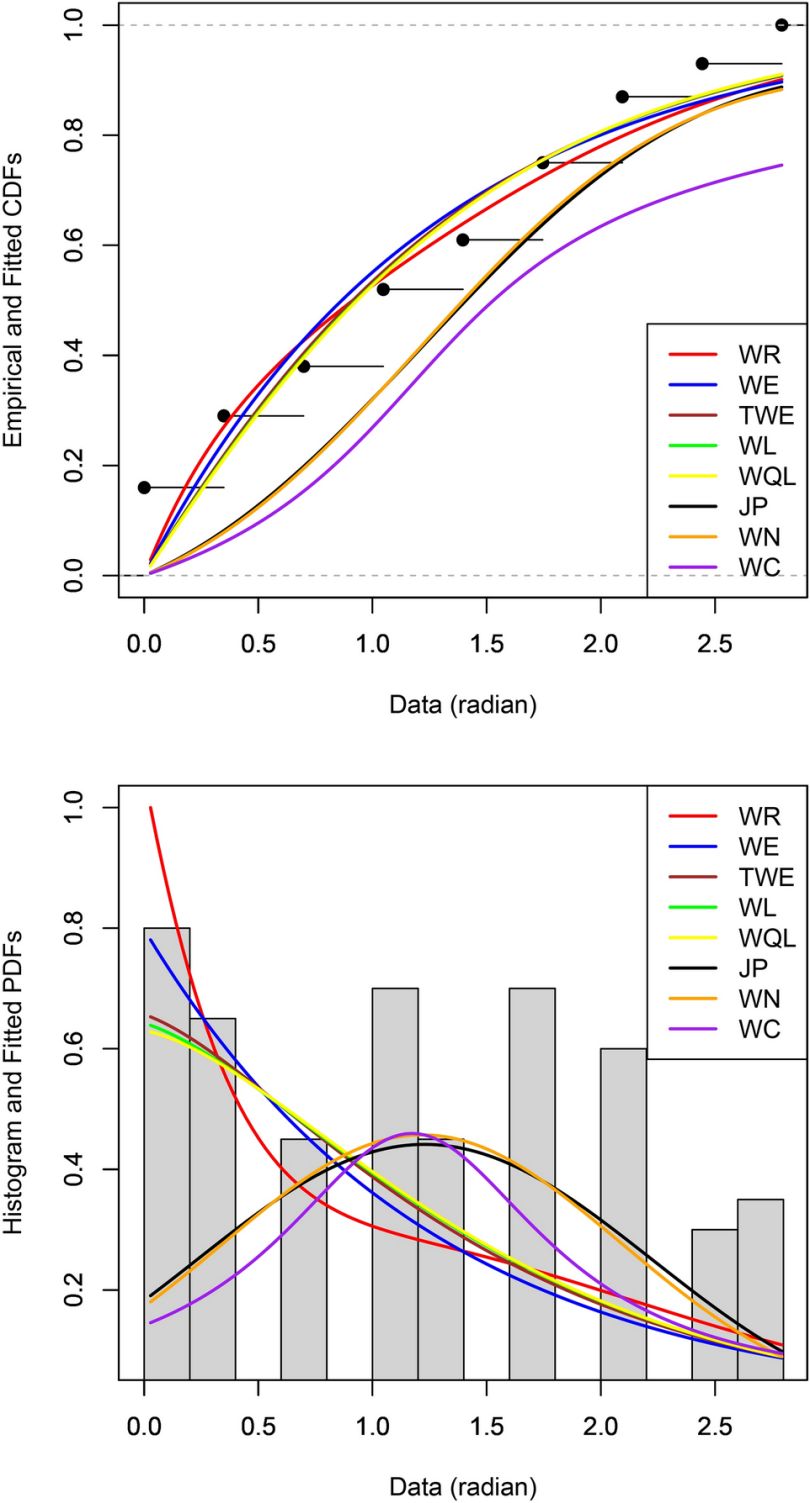
Probability plot (top) and density plot (bottom) of the fitted distributions for the pebbles data set.

The parameter estimates, their standard errors as well as values of the Akaike information criterion (AIC) due to Akaike^1^and the Bayesian information criterion (BIC) due to Schwarz^21^ are given in Tables 1 and 2 for the two data sets. Figures 6 and 7 show the probability plots and density plots of the fitted distributions for the two data sets. The probability plots show the fitted cumulative distribution functions and the empirical cumulative distribution function. The density plots show the fitted probability density functions and histogram of the data.

Sample sizes of the two data sets are 60 and 100, respectively. Given the conclusions reported in Section 5, the standard errors for the first data set in Table 1 should be treated conservatively. The two data sets were recorded in degrees. The data were converted to radians before fitting of the distributions.

### Feldspar laths data set

This data set was initially obtained from Smith (^23^, set 24-6-5) and subsequently published in Fisher (1993, Appendix B5). It comprises of measurements of long-axis orientations for 60 feldspar laths in basalt recorded in degrees.

**Table 1:** MLEs, AICs and BICs for the feldspar laths data set.

Distribution	Parameter estimates (ses)	AIC	BIC
WR	$\widehat{\lambda } = 1.787 (0.108)$	155.965	158.059
WE	$\widehat{\lambda } = 0.664 (0.101)$	161.430	163.524
TWE	$\widehat{\lambda } = 0.832 (0.134)$, $\widehat{\Lambda } = -0.436 (0.247)$	161.448	165.636
WL	$\widehat{\lambda } = 1.031 (0.110)$	158.854	160.948
WQL	$\widehat{\alpha } = 0.713 (0.537)$, $\widehat{\beta } = 1.101 (0.178)$	160.635	164.824
JP	$\widehat{\mu } = 1.436 (0.126)$, $\widehat{\kappa } = 8.187 (71.287)$, $\widehat{\psi } = 0.650 (0.142)$	173.094	179.377
WN	$\widehat{\mu } = 1.407 (0.130)$, $\widehat{\kappa } = 0.987 (0.180)$	175.066	179.254
WC	$\widehat{\mu } = 1.056 (0.183)$, $\widehat{\kappa } = 0.452 (0.069)$	191.752	195.941

**Table 2:** MLEs, AICs and BICs for the pebbles data set.

Distribution	Parameter estimates (ses)	AIC	BIC
WR	$\widehat{\lambda } = 1.931 (0.091)$	226.471	229.076
WE	$\widehat{\lambda } = 0.793 (0.087)$	240.223	242.828
TWE	$\widehat{\lambda } = 0.908 (0.109)$, $\widehat{\Lambda } = -0.279 (0.174)$	240.332	245.543
WL	$\widehat{\lambda } = 1.181 (0.095)$	236.638	239.243
WQL	$\widehat{\alpha } = 1.105 (0.591)$, $\widehat{\beta } = 1.195 (0.147)$	238.623	243.834
JP	$\widehat{\mu } = 1.228 (0.591)$, $\widehat{\kappa } = 9.613 (27.848)$, $\widehat{\psi } = 0.457 (0.732)$	258.381	266.197
WN	$\widehat{\mu } = 1.218 (0.087)$, $\widehat{\kappa } = 1.313 (0.186)$	260.545	265.756
WC	$\widehat{\mu } = 1.173 (0.135)$, $\widehat{\kappa } = 0.486 (0.047)$	297.725	302.936

Table 1 shows that the WR distribution provides the best fit, followed by WL, WE, TWE, JP, WN and WC distributions. The standard error for one of the parameters of the JP distribution is too large. Figure 6 confirms the best fit of the WR distribution.

### Pebbles data set

These data were first documented by Krumbein^12^. Over the years, the data have been referenced by various authors, including Mardia (1972, Table 1.6) and Fisher (1993, Appendix B8). Their values pertain to the horizontal axes of 100 outwash pebbles collected from a late Wisconsin outwash terrace near Cary, Illinois, along the Fox River.

Table 2 shows that the WR distribution provides the best fit, followed by WL, WQL, WE and TWE distributions. The WN distribution gives the second worst fit with respect to AIC. The JP distribution gives the second worst fit with respect to BIC. The WC distribution gives the worst fit with respect to both AIC and BIC. The standard error for one of the parameters of the JP distribution is once again too large. Figure 7 yet again confirms the best fit of the WR distribution.

## Conclusions

We have proposed a one-parameter distribution for circular data. The distribution was constructed by the method of wrapping. Yet the distribution yields elementary expressions for its probability density function, shape properties of the probability density function, cumulative distribution function, trigonometric moments, mean angle, mean resultant, circular variance, circular skewness and circular kurtosis. We have shown that the distribution outperforms the Jones-Pewsey distribution and several other distributions for two data sets in spite of having only one parameter. The Jones-Pewsey distribution has three parameters. Four of the other distributions has two parameters each. The performance of all of the fitted distributions was assessed in terms of AIC values, BIC values, probability plots and density plots.

A future work is to develop an R package for computing the probability density function, cumulative distribution function, trigonometric moments, mean angle, mean resultant, circular variance, circular skewness, circular kurtosis and performing maximum likelihood estimation for the wrapped Rama distribution. Another future work is to generalize the wrapped Rama distribution to bivariate, multivariate, matrix variate and complex variate cases. Yet another future work is to consider other estimation methods for the wrapped Rama distribution, including the method of moments, generalized method of moments, method of weighted moments, method based on minimum distance, method based on minimum mean squared error, method based on maximum a posteriori, method based on maximum entropy, minimum variance unbiased method, best linear unbiased method, least squares method, weighted least squares method, *L* moments method, trimmed *L* moments method, Bayesian methods and the method of probability weighted moments.

## Supplementary Information

Supplementary Information.

## Data Availability

The data can be obtained by contacting the corresponding author.

## Code availability

The code can be obtained by contacting the corresponding author.
