# Supplementary material for: The wrapped Rama distribution
Source: Sci Rep. 2024 Dec 30;14:31936. doi: 10.1038/s41598-024-83436-x (PMC11686034; doi:10.1038/s41598-024-83436-x)
Supplement: Supplementary file 1 — Supplementary Information. [file 41598_2024_83436_MOESM1_ESM.pdf]

Appendix A: R codes

A.1 R codes for Figure 1

```
x=2*pi*seq(0,1,0.001)
a=0.5
A=a**4/((a**3+6)*(1-exp(-2*a*pi)))
B=6*pi*exp(-2*a*pi)/(1-exp(-2*a*pi))
C=2*pi*(1+exp(-2*a*pi))*B/(1-exp(-2*a*pi))
D=1+8*pi**3*exp(-2*a*pi)*(1+4*exp(-2*a*pi)+exp(-4*a*pi))/(1-exp(-2*a*pi))**3
f1=A*exp(-a*x)*(x**3+B*x**2+C*x+D)
a=0.8
A=a**4/((a**3+6)*(1-exp(-2*a*pi)))
B=6*pi*exp(-2*a*pi)/(1-exp(-2*a*pi))
C=2*pi*(1+exp(-2*a*pi))*B/(1-exp(-2*a*pi))
D=1+8*pi**3*exp(-2*a*pi)*(1+4*exp(-2*a*pi)+exp(-4*a*pi))/(1-exp(-2*a*pi))**3
f2=A*exp(-a*x)*(x**3+B*x**2+C*x+D)
a=1
A=a**4/((a**3+6)*(1+exp(-2*a*pi)))
B=6*pi*exp(-2*a*pi)*A/(1-exp(-2*a*pi))
C=2*pi*(1+exp(-2*a*pi))*B/(1-exp(-2*a*pi))
D=(1+8*pi**3*exp(-2*a*pi)*(1+4*exp(-2*a*pi)+exp(-4*a*pi))/(1-exp(-2*a*pi))**3)
f3=A*exp(-a*x)*(x**3+B*x**2+C*x+D)
a=2
A=a**4/((a**3+6)*(1-exp(-2*a*pi)))
B=6*pi*exp(-2*a*pi)/(1-exp(-2*a*pi))
C=2*pi*(1+exp(-2*a*pi))*B/(1-exp(-2*a*pi))
D=1+8*pi**3*exp(-2*a*pi)*(1+4*exp(-2*a*pi)+exp(-4*a*pi))/(1-exp(-2*a*pi))**3
f4=A*exp(-a*x)*(x**3+B*x**2+C*x+D)
a=5
A=a**4/((a**3+6)*(1-exp(-2*a*pi)))
B=6*pi*exp(-2*a*pi)/(1-exp(-2*a*pi))
C=2*pi*(1+exp(-2*a*pi))*B/(1-exp(-2*a*pi))
D=1+8*pi**3*exp(-2*a*pi)*(1+4*exp(-2*a*pi)+exp(-4*a*pi))/(1-exp(-2*a*pi))**3
f5=A*exp(-a*x)*(x**3+B*x**2+C*x+D)
yy=range(f1,f2,f3,f4,f5)
xx=c(0,2*pi)

yy[2]=0.5
plot(x,f1,xlab=expression(theta),ylab="PDF",type="l",xlim=xx,ylim=yy,lwd=2)
par(new=TRUE)
plot(x,f2,xlab="",ylab="",type="l",xlim=xx,ylim=yy,lwd=2,col="red")
par(new=TRUE)
plot(x,f3,xlab="",ylab="",type="l",xlim=xx,ylim=yy,lwd=2,col="blue")
par(new=TRUE)
plot(x,f4,xlab="",ylab="",type="l",xlim=xx,ylim=yy,lwd=2,col="brown")
par(new=TRUE)
```

```
plot(x,f5,xlab="",ylab="",type="l",xlim=xx,ylim=yy,lwd=2,col="green")
legend("topright",legend=c(expression(lambda==0.5),expression(lambda==0.8),
expression(lambda==1),expression(lambda==2),expression(lambda==5)),
col=c("black","red","blue","brown","green"),lty=1,lwd=2)
```

A.2 R codes for Section 5

```
a=1
aa=a
F=function (x)
{tt=x
A=a**4/((a**3+6)*(1-exp(-2*a*pi)))
B=6*pi*exp(-2*a*pi)/(1-exp(-2*a*pi))
C=2*pi*(1+exp(-2*a*pi))*B/(1-exp(-2*a*pi))
D=1+8*pi**3*exp(-2*a*pi)*(1+4*exp(-2*a*pi)+exp(-4*a*pi))/(1-exp(-2*a*pi))**3
ff=function (y) {A*exp(-a*y)*(y**3+B*y**2+C*y+D)}
for (i in 1:length(x)) {tt[i]=integrate(ff,lower=0,upper=x[i])$value}
return(tt)}

nsim=1000
aest=rep(0,nsim)
len=aest

bias=rep(0,91)
mse=bias
cl=bias
cp=bias

for (n in seq(10,100))
{jj=0
for (ii in seq(1,nsim))
{

u=runif(n,min=0,max=1)
xx=u
for (i in seq(1,n))
{FF=function (yy) {F(yy)-u[i]}
xx[i]=uniroot(FF,lower=0,upper=(2*pi))$root}


f=function (p)
{a=p[1]
tt=1.0e20
if (a > 0)
{A=a**4/((a**3+6)*(1-exp(-2*a*pi)))
B=6*pi*exp(-2*a*pi)/(1-exp(-2*a*pi))
```

```
C=2*pi*(1+exp(-2*a*pi))*B/(1-exp(-2*a*pi))
D=1+8*pi**3*exp(-2*a*pi)*(1+4*exp(-2*a*pi)+exp(-4*a*pi))/(1-exp(-2*a*pi))**3
tt=-n*log(A)+a*sum(xx)-sum(log(xx**3+B*xx**2+C*xx+D))}
return(tt)}

est=optim(f,par=c(1),method="Brent",lower=0,upper=(2*pi),hessian=TRUE)

len[ii]=NA
if (est$hessian>0)
{len[ii]=2*qnorm(0.975)*sqrt(1/est$hessian)
if (aa<est$par+0.5*len[ii]&aa>est$par-0.5*len[ii]) jj=jj+1}
aest[ii]=est$par}

bias[n-9]=mean(aest-aa)
mse[n-9]=mean((aest-aa)**2)
cl[n-9]=mean(len[!is.na(len)])
cp[n-9]=jj/nsim}


plot(seq(10,100),bias,xlab="n",ylab="Bias",type="l",lwd=2)
abline(h=0,col="red",lwd=2)

plot(seq(10,100),mse,xlab="n",ylab="Mean squared error",type="l",lwd=2)
abline(h=0,col="red",lwd=2)

plot(seq(10,100),cl,xlab="n",ylab="Coverage length",type="l",lwd=2)
abline(h=0,col="red",lwd=2)

plot(seq(10,100),cp,xlab="n",ylab="Coverage probability",type="l",lwd=2)
abline(h=0.95,col="red",lwd=2)
```

A.3 R codes for Section 6

```
#WR
library(bbmle)
library(circular)
x=fisherB5*pi/180
dL <- function(x,lambda) {
k <- 0:1000
r <- max( sum(lambda^4/(6+lambda^3)*(1+(x+2*k*pi)^3)*exp(-
lambda*(x+2*k*pi))) , 0.00000001)
}
vdL <- Vectorize(dL)
LL <- function(x,lambda){ -sum( log( vdL(x,lambda))) }
(m0 <- mle2(LL,start=list(lambda=1.7),data=list(x=x)))
summary(m0)
```

```
xx=min(x)+(max(x)-min(x))*seq(0.01,1,0.01)
lambda=1.78671
d1=vdL(xx,lambda)

#WE
library(bbmle)
library(circular)
x=fisherB5*pi/180
dL <- function(x,lambda) {
k <- 0:1000
r <- max( sum(lambda*exp(-lambda*(x+2*k*pi))*as.numeric(lambda>0)) ,
0.00000001)
}
vdL <- Vectorize(dL)
LL <- function(x,lambda){ -sum( log( vdL(x,lambda))) }
(m0 <- mle2(LL,start=list(lambda=0.6),data=list(x=x)))
summary(m0)

lambda=0.66400
d2=vdL(xx,lambda)


#TWE
library(bbmle)
library(circular)
x=fisherB5*pi/180
dL <- function(x,lambda,Lambda) {
k <- 0:1000
r <- max(2*lambda*Lambda*exp(-lambda*(x+2*k*pi))*(exp(-
lambda*(x+2*k*pi))-1)/(exp(-2*pi*lambda)-1)^2 - lambda/
(exp(-2*pi*lambda)-1)*(Lambda+1)*exp(-lambda*(x+2*k*pi))*as.numeric(lambda>0),
0.00000001)
}
vdL <- Vectorize(dL)
LL <- function(x,lambda,Lambda){ -sum( log( vdL(x,lambda,Lambda))) }
(m0 <- mle2(LL,start=list(lambda=0.8,Lambda=-0.4),data=list(x=x)))
summary(m0)

lambda=0.83220
Lambda=-0.43586
d3=vdL(xx,lambda,Lambda)

#WL
library(bbmle)
library(circular)
x=fisherB5*pi/180
dL <- function(x,lambda) {
k <- 0:1000
```

```
r <- max( sum(lambda^2/(1+lambda)*(1+x+2*k*pi)*exp(-
lambda*(x+2*k*pi)))*as.numeric(lambda>0) , 0.00000001)
}
vdL <- Vectorize(dL)
LL <- function(x,lambda){ -sum( log( vdL(x,lambda))) }
(m0 <- mle2(LL,start=list(lambda=1.03),data=list(x=x)))
summary(m0)

lambda=1.03085
d4=vdL(xx,lambda)

#WQL
library(bbmle)
library(circular)
x=fisherB5*pi/180
dL <- function(x,alpha,beta) {
k <- 0:1000
r <- max( sum(beta/(alpha+1)*(alpha+beta*(x+2*k*pi))*exp(-
beta*(x+2*k*pi))), 0.00000001)
}
vdL <- Vectorize(dL)
LL <- function(x,alpha,beta){ -sum( log( vdL(x,alpha,beta))) }
(m0 <- mle2(LL,start=list(alpha=0.7,beta=1.1),data=list(x=x)))
summary(m0)

alpha=0.71271
beta=1.10061
d5=vdL(xx,alpha,beta)

#JP

vdL <- Vectorize(dJp)
LL <- function(x,mu,kappa,psi){ -sum( log( vdL(x,mu,kappa,psi))) }
(m0 <- mle2(LL,start=list(mu=0.7,kappa=1.1,psi=1),data=list(x=x)))
summary(m0)

mu=1.43593
kappa=8.86475
psi=0.64985
d6=dJp(xx,mu,kappa,psi)


install.packages("BAMBI")
library(BAMBI)
ff=function (p)
{tt=1.0e20
if (p[2]>0) tt=-sum(dwnorm(x,kappa=p[2],mu=p[1],log=TRUE))
return(tt)}
```

```
est=optim(ff,par=c(0,1),hessian=TRUE)
d99=dwnorm(xx,kappa=est$par[2],mu=est$par[1])

ff=function (p)
{tt=1.0e20
if (p[2]>0&p[2]<1) tt=-sum(log(dwrpcauchy(x,mu=p[1],rho=p[2])))
return(tt)}
est=optim(ff,par=c(0,0.1),hessian=TRUE)
d999=dwrpcauchy(xx,mu=est$par[1],rho=est$par[2])


xx2=range(x)
yy=range(d1,d2,d3,d4,d5,d6,d99,d999)
hist(x,freq=F,nclass=10,xlab="",ylab="",main="",xlim=xx2,ylim=yy)
par(new=TRUE)
plot(xx,d1,type="l",xlab="",ylab="",lty=1,lwd=2,col="red",xlim=xx2,ylim=yy)
par(new=TRUE)
plot(xx,d2,type="l",xlab="",ylab="",lty=1,lwd=2,col="blue",xlim=xx2,ylim=yy)
par(new=TRUE)
plot(xx,d3,type="l",xlab="",ylab="",lty=1,lwd=2,col="brown",xlim=xx2,ylim=yy)
par(new=TRUE)
plot(xx,d4,type="l",xlab="",ylab="",lty=1,lwd=2,col="green",xlim=xx2,ylim=yy)
par(new=TRUE)
plot(xx,d5,type="l",xlab="",ylab="",lty=1,lwd=2,col="yellow",xlim=xx2,ylim=yy)
par(new=TRUE)
plot(xx,d6,type="l",xlab="",ylab="",lty=1,lwd=2,col="black",xlim=xx2,ylim=yy)
par(new=TRUE)
plot(xx,d99,type="l",xlab="",ylab="",lty=1,lwd=2,col="orange",xlim=xx2,ylim=yy)
par(new=TRUE)
plot(xx,d999,type="l",xlab="",ylab="",lty=1,lwd=2,col="purple",xlim=xx2,ylim=yy)
title(xlab="Data (radian)",ylab="Histogram and Fitted PDFs")
legend("topright",legend=c("WR","WE","TWE","WL","WQL","JP","WN","WC"),
col=c("red","blue","brown","green","yellow","black","orange","purple"),lty=1,lwd=2)
```
